# Effects of Fumaria parviflora leaves extract on reproductive parameters in adult male rats 

**Published:** 2013-11

**Authors:** Mehran Dorostghoal, Seyyed Mansoor Seyyednejad, Lotfollah Khajehpour, Ayoob Jabari

**Affiliations:** *Department of Biology, Faculty of Science, Shahid Chamran University of Ahvaz, Ahvaz, Iran.*

**Keywords:** *Fumaria parviflora Lam.*, *Male fertility*, *Spermatogenesis*, *Sperm quality*, *Testosterone*

## Abstract

**Background:** There is growing concern that occupational, environmental and lifestyle factors adversely affect male reproductive health. *Fumaria parviflora Lam*. is being used traditionally in Persian folk medicine to cure various ailments and has been supposed to have fertility-enhancing properties.

**Objective: **A dose-response study was designed to assess effects of *F. parviflora* ethanolic leaves extract on reproductive parameters in adult male Wistar rats.

**Materials and Methods:** In this experimental study, healthy adult male rats were treated with 100, 200 and 400 mg/kg/day of *F. parviflora* leaves extract via gavage for 70 days. Blood samples were collected for determination of testosterone, LH and FSH serum levels. Reproductive organs weight, motility, morphology and density of epididymal sperm, seminiferous tubules diameter and germinal epithelium height were evaluated in each experimental group.

**Results: **The body weight was not affected, while the weights of testis and epididymis were significantly enhanced in rats treated with 200 and 400 mg/kg/day *F. parviflora* extract. No significant changes were observed in seminal vesicle and ventral prostate weight between experiment groups. Significant increase was found in epididymal sperm density and percent of morphologically normal sperm in extract-treated rats. Serum testosterone levels were significantly higher in rats received 200 and 400 mg/kg/day.

**Conclusion:** The results indicated that ethanolic extract of *F. parviflora* leaves have a potential to improve reproductive parameters and enhance male fertility.

## Introduction

Current evidence suggests human sperm quality has been deteriorated due to environmental and occupational exposure to various chemicals, heat, radiation, and heavy metals (1, 2). In addition, it has been reported that lifestyle risk factors such as cigarette smoking, alcohol consumption, chronic stress and nutritional deficiencies could adversely affect the spermatogenesis (3, 4). Recently, the role of oxidative stress has been suggested in the pathogenesis of male infertility (5). 

Oxidative stress can arise as a consequence of excessive production of reactive oxygen species (ROS) or impaired antioxidant defense mechanisms (6). So, it has been claimed that protective agents against ROS, such as antioxidants, may be useful therapeutic agents for male infertility (7, 8). In this regard, plants have a long folklore of use in aiding fertility, including fertility-enhancing properties and aphrodisiacal qualities (9). 

*Fumaria parviflora *Lam. (Fumariaceae) is an herbaceous plant that grows in a wide variety parts of Iran, Indo-Pakistan subcontinent and Turkey. It is known as Shahtareh in Iran and aerial parts of* F. parviflora *has been used traditionally in Iranian folk medicine against liver and bile duct disorders, as diuretic and laxative and for its male fertility-promoting properties (10-13). It is also considered for treatment of hepatobiliary disorders, dermatological diseases, anthelmintic, antipyretic, and as a blood purifier (14-18). 

Phytochemical analyses of some plants of genus *Fumaria*, including *F. parviflora *has indicated presence of isoquinoline alkaloids namely protropine, cryptopine, sinactine, stylopine, bicuculine, adlumine, parfumine, fumariline, fumarophycine, fumaritine, dihydrofumariline, perfumidine and dihydrosanguirine (19). It has been reported that *F. parviflora* and *F. vaillantii* another species of genus *Fumaria*, have ability to counteract CCl_4_ induced hepatotoxicity with its antioxidative properties (20, 21). 

Protective effect of *F. parviflora *against paracetamol-induced hepatotoxicity and its beneficial effects in the treatment of the hepatobiliary system diseases have also been demonstrated (22, 23). Soušek *et al* reported that phenolic extracts from *Fumaria* were 2-3 folds more effective as scavengers of 1,1-diphenyl-2-picrylhydrazyl radical than the alkaloid extracts (24). Species and tissue-specificity of prokinetic, laxative and spasmodic effects of *F. parviflora* aqueous-methanol extract have been shown (25). It has been indicated that *F. parviflora* has significant hypoglycemic effect on streptozotocin-induced diabetic rats (26). 

Anti-nociceptive effect of *F. parviflora* percolated extract in mice and rats and its beneficial effect against hand eczema have been shown (27, 28). Moreover, it was shown that* F. parviflora* has favorable effect on spermatogenesis in male rats (13). In addition, anti-inflammatory activities of *F. parviflora* and* Fumaria indica*, another species of genus *Fumaria*, have been reported (29, 30).

Therefore, present study was undertaken to evaluate the impacts of varying doses of *F. parviflora* ethanolic leaves extract on the reproductive parameters in adult Wistar male rats.

## Materials and methods

**Plant materials**

The leaves of *F. parviflora *were collected from Kazeroun city in Fars province of Iran in March 2012. It was recognized and authenticated in Botanical Systematic Laboratory, Department of Biology, Shahid Chamran University of Ahvaz, Iran.

**Preparation of ethanolic extract of** ***F. parviflora***

Fresh leaves of *F. parviflora* were dried at 40^o^C for 48 hours. The dried leaves (200 g) were powdered in an electrical grinder and extracted successively with ethanol (600 ml) in a Soxhlet extractor for 48h at 60^o^C. After extraction, the mixture was filtered and the solvent was evaporated to dryness at 50-55^o^C by using a rotary evaporator and the extract left behind (yield was 5.6 g/kg) was stored at 4^o^C. It was dissolved in distilled water whenever needed for experiments.

**Animals**

In this experimental study, forty healthy adult male Wistar rats (8 weeks old, 180-200 g body weight) were obtained from Research Center and Experimental Animal House of Ahwaz Jundishapour University of Medical Sciences. The Animals were housed under standard laboratory conditions (temperature 23±2^o^C, relative humidity of 50±5%, 12h/12h light/dark cycle) and were given food and water *ad libitum*. The Animal Ethics Committee of Shahid Chamran University of Ahvaz has approved the experimental protocol. 

**Experimental design**

After one week of acclimatization, animals were divided randomly into four equal groups (n=10) as follows: 

Group 1 (control), was administered 1 ml distilled water via gavage once daily for 70 days. 

Group 2, was administered with ethanolic extract of *F. parviflora *at dose of 100 mg/kg body weight via gavage once daily for 70 days. Male rats were exposed to *F. parviflora *extract for 70 days.

Group 3, was administered with ethanolic extract of *F. parviflora *at dose of 200 mg/kg body weight via gavage once daily for 70 days. 

Group 4, was administered with ethanolic extract of *F. parviflora *at dose of 400 mg/kg body weight via gavage once daily for 70 days. 

The body weight of rats was recorded at the termination of the experiment. After the administration of the last treatment, the animals were fasted overnight and then on the next day, they were sacrificed under light ether anesthesia. 

Blood samples were collected by heart puncture for determination of testosterone, luteinizing hormone and follicle-stimulating hormone levels. The reproductive organs; testes, epididymis, seminal vesicles and ventral prostate were removed and weighed. The testes immediately fixed into Bouin’s solution for morphometrical study.

**Morphometrical analysis**

Tissue samples from right testes were excised, and processed for paraffin embedding sections. Serial sections with 5μm thickness were stained with heamatoxylin and eosin and used for morphometrical studies at light microscopic level. For measuring of seminiferous tubules diameter and germinal epithelium height, 90 round or nearly round cross-sections of seminiferous tubules were randomly chosen in each rat. 

Then, using an ocular micrometer of light microscopy (Olympus BH, Japan, Tokyo), at a magnification of ×40, two perpendicular diameters of each cross-section of seminiferous tubules were measured and the mean of these was calculated. Also, germinal epithelium height in 4 equidistance of each cross-section of seminiferous tubules measured and the mean of these was calculated (31).

**Epididymal sperm analysis**

Epididymis was separated carefully from testis and divided into 3 segments; head, body and tail. The epididymal tail was trimmed with scissors and placed in petri dishes containing 1.0 ml of 0.1 M phosphate buffer of pH 7.4. The *dish*es *gently* swirled for homogeneity and allowed sperm diffusion in the solution for 10 min under 37^o^C for dispersion of sperm cells. Sperm samples were assessed for motility, number and gross morphology of sperms without the investigator knowing which samples were from which group. 

For analysis of sperm motility an aliquot of 10 μL was placed in a hemocytometer chamber (Paul Marienfeld GmbH,** Lauda-Königshofen,** Germany) and analyzed under a light microscope. One hundred sperm were evaluated per animal and classified into motile and immotile (32). For evaluation of sperm density, an hour after the sperm diffusion in the solution a 10 μL aliquot of the epididymal sperm suspension was transferred to each counting chamber of the hemocytometer and allowed to stand for 5 min. The cells which settled during this time were counted by a light microscope at ×40 magnification. The sperm heads were counted and expressed as million/ml of suspension (32). 

The sperm morphology was also determined using eosin staining method. For this purpose 10 μl of 1% eosin Y was added to a test tube containing 40 μl of sperm suspension and were mixed by gentle agitation. Then, sperm were incubated at room temperature for 45-60 min for staining and then re-suspended with a Pasteur pipette. Two hundred sperm per animal were examined microscopically at ×40-100 magnifications and the number of morphologically abnormal sperm was recorded to give the percent abnormal sperm.

**Serum hormonal assay**

Blood samples were left for 60 min to clot and then centrifuged for 10 min at 2430×g. The obtained clear sera were stored at -80^o^C until testosterone, luteinizing hormone and follicle-stimulating hormone levels were measured by radioimmunoassay using commercial kit (AccuBind ELISA Kits, California, USA). Analyses were carried out according to the manufacturer’s instructions.

**Statistical analysis**

The data were analyzed using the Statistical Package for Social Science program version 10 (SPSS 10). Statistical analysis was done using analysis of variance (ANOVA) followed by Tukey’s test. Data are expressed as the mean±SD and the level of significance was set as p<0.05.

## Results

**Clinical signs of toxicity**

All the animals in any of the control and *F. parviflora *extract-treated groups were apparently normal and did not show any visible signs of toxicity.

**Body and reproductive organs weight**

There were no statistically significant differences in the body weight of the animals between extract-treated and control groups. While significant (p<0.05) increases were seen in the testis and epididymis weights of male rats treated with 200 and 400 mg/kg *F. parviflora *extract in compared with control group. 

The treatment of rats with *F. parviflora *extract caused no significant differences in weight of ventral prostate and seminal vesicle between extract-treated and control groups (Table I).

**Morphometrical parameters**

In male rats treated with 200 and 400 mg/kg *F. parviflora* extract seminiferous tubules diameter and germinal epithelium height were higher significantly (p<0.05) in compared with control group (Table II).

**Sperm parameters**

Significant (p<0.05) dose-related increase in sperm density was observed in* F. parviflora* extract-treated rats in compared with control group. Also, *F. parviflora *administration to adult male rats after 70 days caused a significant (p<0.05) dose-related decrease in the percentage of morphologically abnormal sperm. There were no significant differences in percentage of motile sperm between *F. parviflora *extract-treated and control groups (Table II).

**Serum levels of hormones**

No statistically significant differences were observed in serum FSH and LH levels between *F. parviflora *extract-treated and control groups. Testosterone levels increased significantly (p<0.05) in rats treated with 200 and 400 mg/kg *F. parviflora *extract as compared to control group (Table III).

**Table I:** Effects of *F. parviflora* extract on body and reproductive organs weight in adult male Wistar rats

**P** **a** **r** **am** **ete** **rs**	**Control**	***F*** **.** *** parviflora*** **(100 mg/kg)**	***F*** **.** *** parviflora*** **(200 mg/kg)**	***F*** **.** *** parviflora*** **(400 mg/kg)**	**p-value** *
Body (g)	335.1 ± 2.1	333.3 ± 4.3	338.7 ± 2.2	340.5 ± 4.1	1.000
Testis (mg)	1364.5 ± 12.4	1373.1 ± 15.2	1462.6 ± 13.1^a^	1475.2 ± 14.2^a^	0.032
Epididymis (mg)	463.5 ± 4.6	469.3 ± 5.2	530.2 ± 4.4^a^	541.4 ± 4.3^a^	0.027
Seminal vesicle (mg)	450.8 ± 4.1	452.6 ± 5.3	460.4 ± 4.3	463.3 ± 5.1	0.910
Ventral prostate (mg)	208.8 ± 2.4	212.2 ± 3.4	210.4 ± 3.0	215.8 ± 3.2	1.000

* ANOVA followed by Tukey’s test. Data are presented as mean±SD.

a : Differ significantly (p<0.05) respect to control rats.

**Table II:** Effects of *F. parviflora *extract on seminiferous tubule and sperm parameters in adult male Wistar rats

**P** **a** **r** **am** **ete** **rs**	**Control**	***F*** **.** *** parviflora*** **(100 mg/kg)**	***F*** **.** *** parviflora*** **(200 mg/kg)**	***F*** **.** *** parviflora*** **(400 mg/kg)**	**p-value** *
STD (μm)	249.2 ± 2.1	252.5 ± 3.0	268.3 ± 3.5^a^	271.5 ± 2.0^a^	0.038
GEH (μm)	65.7 ± 1.7	69.5 ± 3.8	76.3 ± 2.6^a^	79.5 ± 2.4^a^	0.020
Sperm density (million/ml)	65.81 ± 2.3	72.70 ± 3.0^a^	75.23 ± 2.4^a^	78.05 ± 2.8^a^	0.002
Motile sperm (%)	78.52 ± 2.5	80.36 ± 2.4	80.31 ± 3.1	81.72 ± 4.1	0.921
Abnormal sperm (%)	16.6 ± 0.62	14.2 ± 0.53	8.8 ± 0.72^a^	8.9 ±0.51^a^	0.007

STD: Seminiferous tubule diameter; GEH: Germinal epithelium height.

* ANOVA followed by Tukey’s test. Data are presented as mean±SD.

a : Differ significantly (p<0.05) respect to control rats.

**Table III:** Effects of *F. parviflora *extract on serum testosterone, LH and FSH levels in adult male Wistar rats

**P** **a** **r** **am** **ete** **rs**	**Control**	***F*** **.** *** parviflora*** **(100 mg/kg)**	***F*** **.** *** parviflora*** **(200 mg/kg)**	***F*** **.** *** parviflora*** **(400 mg/kg)**	**p-value** *
Testosterone (ng/ml)	2.52 ± 0.50	2.84 ± 0.44	4.50 ± 0.73^a^	5.28 ± 0.80^a^	0.012
LH (ng/ml)	0.68 ± 0.04	0.62 ± 0.02	0.71 ± 0.03	0.65 ± 0.03	0.883
FSH (ng/ml)	2.25 ± 0.02	2.10 ± 0.03	2.21 ± 0.02	2.14 ± 0.04	1.000

* ANOVA followed by Tukey’s test. Data are presented as mean±SD.

a : Differ significantly (p<0.05) respect to control rats.

## Discussion

*F. parviflora *has been well known for its medicinal properties in wide variety areas of world and is supposed to have fertility-enhancing properties in Iranian folk medicine (10-13). In the present study, effects of* F. parviflora *leaves extract on the reproductive parameters were evaluated in adult Wistar male rats. The results demonstrate that *F. parviflora *leave extract increase testis and epididymis weights, sperm with normal morphology, sperm counts and serum testosterone levels in adult male rats. 

Thus,* F. parviflora *leave extract has beneficial effects on male reproductive function in adult male rats. A similar finding was reported by Heydari Nasrabadi *et al*, who indicated that* F. parviflora *has positive effect on male reproductive system. They administered *F. parviflora *orally at doses of 750 and 1050 mg/kg for 3 days and 250 mg/kg for 5 days by gavage. Heydari Nasrabadi *et al* reported that *F. parviflora* significantly (p<0.001) increased the number of spermatogonium, spermatocytes, spermatozoids and Leydig cells (13).

However, in present study male rats were exposed to *F. parviflora* extract for 70 days in order to evaluate its effect through a complete spermatogenic cycle (33). It has been considered that the structural and functional integrity of reproductive organs depends on the adequate bioavailability of testosterone (34). 

Androgens have been shown to be necessary for the development, growth and normal functioning of the testes and male accessory reproductive glands and studies have shown that the level of testosterone is positively correlated with the weights of testis and epididymis (35). Therefore, the significant increase in testis and epididymis weight could be due to increased androgen biosynthesis as evidenced by a significant increase in serum testosterone levels in the extract-treated rats. 

In this regard, Heydari Nasrabadi *et al* reported that* F. parviflora *extract increased the number of Leydig cells in male rats. They also concluded that increase in spermatogenic cells number is due to increase in angiogenesis and more blood flow to the testis (13). Furthermore, in present study dose-related increases were observed in sperm count in *F. parviflora *extract-treated rats. One of the most sensitive tests for evaluating spermatogenesis is sperm count in the epididymis (36). However, number of stored sperm determines the weight of epididymis. Sperm count was often used as a measure of sperm production, testicular function and/or male fertility. Low sperm count and high percentage of abnormal spermatozoa each have been associated with reduced fertility (37). 

According to our findings the beneficial effects of *F. parviflora *may be due to the improvement of testicular oxidative status by its extract components. Agarwal *et al* reported that increased formation of ROS is correlated with the reduction of sperm motility (38). Thus, the increase in sperm count and percentage of normal morphological sperm thereby shows that treatment with *F. parviflora *improves and enhances the fertilizing capacity of semen. Antioxidant components such as isoquinoline alkaloids, phenolic compounds and flavonoids have been reported in *Fumaria *species (39, 40). 

Heydari Nasrabadi *et al* suggested that improved fertility observed in male rats might be due to the antioxidant effect of *F. parviflora *(13). Sousek *et al* found that *Fumaria *species has the ability to counteract oxidative stress with its antioxidative properties (41). Inhibition of arthrosclerosis development by *F. vaillantii* hydroalcoholic extract in rabbit has been related to antioxidant effects of its flavonoids like rutin (42). 

Phytochemical analyses showed that* Fumaria *species contains high amounts of isoquinoline alkaloids including fumaric acid (28). It has been determined that fumaric acid content of *F. parviflora *to be about 0.93% w/w (43). So, isoquinoline alkaloids are found to be the major contributors to the antioxidative activity in *F. parviflora*. Rao and Mishra also suggested that fumaric acid is probably responsible for the antioxidative activity of *F*.* indica *(44). 

It has been shown that fumarates may activate the Nrf2 transcriptional pathway which is known to mediate induction of phase 2 genes by SH-reactive electrophiles and to play a major role in cell and tissue defense against oxidative stress (45).

## Conclusion

It is concluded that the components of the *F. parviflora* leaves extract have the potential to improve male reproductive function and consequently promote fertility which might be a consequence of both its potent antioxidant properties and androgenic activities. Our findings support the traditional use of *F. parviflora* as solution for male reproductive problems. However, the mechanism of the therapeutic efficacy and toxicity of isolated compounds of *F. parviflora* needs to be investigated in future studies. 
